# Association between the lowest level of serum albumin during hospitalization and adverse outcomes in older adults with COVID-19

**DOI:** 10.1097/MD.0000000000040734

**Published:** 2024-11-29

**Authors:** Qiaoli Liu, Haifeng Miao, Chunwei Shi, Piao Hu, Suhong An

**Affiliations:** a Department of Infectious Diseases, Xiaoshan Affiliated Hospital of Wenzhou Medical University, Hangzhou, Zhejiang, China; b Department of Radiotherapy, Xiaoshan Affiliated Hospital of Wenzhou Medical University, Hangzhou, Zhejiang, China.

**Keywords:** albumin, association, COVID-19, outcome, predictive

## Abstract

Serum albumin on admission has been investigated among inpatients with COVID-19. However, studies on the lowest level of serum albumin during hospitalization and adverse outcomes are limited. This research aimed to explore association between them in older adults with COVID-19. A retrospective study was conducted with 300 patients aged 60 or older with first confirmed COVID-19 from January to February 2023. An adverse outcome was defined as development of acute respiratory failure, shock, or death. Data on demographics, comorbidities, laboratory parameters, the initial phase of COVID-19, coinfection, sepsis, receipt of antiviral treatment and outcomes were gathered from the electronic medical records. The association between the lowest level of serum albumin and adverse outcomes was analyzed using univariate and multivariate regression models, along with generalized additive models. After adjusting potential confounders, nonlinear relationship with an inflection point of 29.1 g/L was detected between the lowest level of serum albumin and adverse outcomes in the elderly. The effect sizes and the confidence intervals on the left and right sides of the inflection point were 0.667 (0.520, 0.856) and 1.171 (0.875, 1.568), respectively. This demonstrated that the lowest level of serum albumin was negatively correlated with adverse outcomes when albumin was <29.1 g/L. A rise of 1 unit in the lowest level of albumin equated to a 33.3% decrease in the risk of adverse outcomes. The correlation between the lowest level of serum albumin and adverse outcomes of COVID-19 is a nonlinear. this study indicates that serum albumin levels should be sustained above the critical inflection point identified to reduce the risk of adverse outcomes.

## 1. Introduction

Coronavirus disease 2019 (COVID-19) is a disease caused by severe acute respiratory syndrome coronavirus-2 (SARS-CoV-2) firstly appeared in China in December 2019.^[1]^ Over 635 million infections have been confirmed and more than 6.5 million deaths have occurred just in the last 3 years due to its outbreak.^[2]^ Currently, the continuous cycles of infections persist in posing threats to human health and lives, especially for the elderly.

COVID-19 unusually has a disproportionate impact on those over 50s, whereas children and young adults are less susceptible to it.^[3]^ Even if infected with SARS-CoV-2, young people usually suffer mild or remain asymptomatic, while the elderly are likely to experience more severe symptoms and unfavorable outcomes.^[4,5]^ Previous research shows that the majority of the deceased are aged 60s or older.^[6]^ Given the high mortality rate, it is crucial to identify predictors of adverse outcomes.

Albumin makes up roughly 50% of the synthesized proteins in the liver, amounting to about 14 to 17 g/day, and performs various physiological roles in the body.^[7]^ Nonetheless, decline of albumin has been repeatedly addressed in COVID‐19.^[8–10]^ Furthermore, the predictive value of albumin on admission has also been explored.^[11–13]^ However, it is unclear whether the lowest level of serum albumin during a hospital stay can predict the outcome of COVID-19.

This study aims to explore the association between the lowest level of serum albumin during hospitalization and adverse outcomes in the elderly with COVID‐19.

## 2. Methods

### 2.1. Study population

This observational study was conducted on patients admitted to Xiaoshan Affiliated Hospital of Wenzhou Medical University, Hangzhou city, Zhejiang Province, China, from January 1, 2023, to February 28, 2023. Participants were first diagnosed with SARS-CoV-2 infection by reverse transcription polymerase chain reaction (RT-PCR) performed by the hospital-based clinical laboratory. We followed up patients via the phone until adverse outcomes occurred or 2 weeks after discharge, whichever came first.

Inclusion criteria for patients were as follows: age >60 years; an initial diagnosis of SARS-CoV-2 infection; provision of informed consent.

Conversely, exclusion criteria ruled out patients who had been diagnosed with COVID-19 more than once; were outpatients; were under the age of 60 years; did not provide informed consent.

### 2.2. Definition

An adverse outcome was defined as development of acute respiratory failure, shock, or death during hospitalization. Acute respiratory failure is characterized by critically low arterial oxygen levels (PaO_2_ < 60 mm Hg), and/or elevated arterial carbon dioxide levels (PaCO_2_ > 50 mm Hg), when breathing air at rest. Clinical symptoms, a systolic blood pressure of <90 mm Hg, a reduction of more than 30% from baseline, or a pulse pressure difference of more than 20 mm Hg are defined as shock.

### 2.3. Data collection

All data were collected through electronic records. We extracted demographic information (age, sex, and smoking status), comorbidities data (diabetes mellitus, hypertension, cardiovascular disease, chronic lung disease, and cerebrovascular disease). The lowest values of serum albumin during hospitalization, the corresponding globulin levels and laboratory data on admission were collected including white blood cell count (WBC), lymphocyte count (L), hemoglobin (Hb), platelet count (PLT), C-reactive protein (CRP), D-dimer, alanine aminotransferase (ALT), blood urea nitrogen (BUN), serum creatinine (SCR), and creatine kinase-myocardial isoenzyme (CK-MB). Furthermore, we collected data on coinfection, sepsis, initial COVID-19 stage, antiviral treatment and clinical outcomes (acute respiratory failure, shock, and death).

### 2.4. Statistical analysis

Continuous variables of normal distributions are expressed as mean ± SD, while those of skewed distributions are represented by the median (Q1–Q3). Categorical variables were expressed as n (%). The 1-way ANOVA test was used for normal distribution, Kruskal–Wallis *H* test for skewed distribution, and Chi-square test for categorical variables to determine the statistical differences between groups. Univariate and multivariate regression models and generalized additive models were used to analyze the relationship between the lowest level of albumin and adverse outcomes. Adjustment of covariates adhered to the following principle: inclusion in this model if they altered the matched odds ratio by at least 10%^[14]^; or if existing research has demonstrated that the covariates are independently associated with adverse outcomes.

In addition, if the nonlinear correlation was observed, a 2-piecewise linear regression model was performed to calculate the threshold effect of the lowest value of serum albumin on adverse outcomes in terms of the smoothing plot. When the ratio between adverse outcomes and the lowest value of serum albumin appeared obvious in a smoothed curve, the recursive method automatically calculates the inflection point, where the maximum model likelihood will be used. Statistical analyses were conducted using R (http://www.R-project.org, The R Foundation) and EmpowerStats (http://www.empowerstats.com, X&Y Solutions, Inc., Boston, MA). *P* values <.05 (2-sided) were considered statistically significant.

## 3. Results

**Figure 1.**
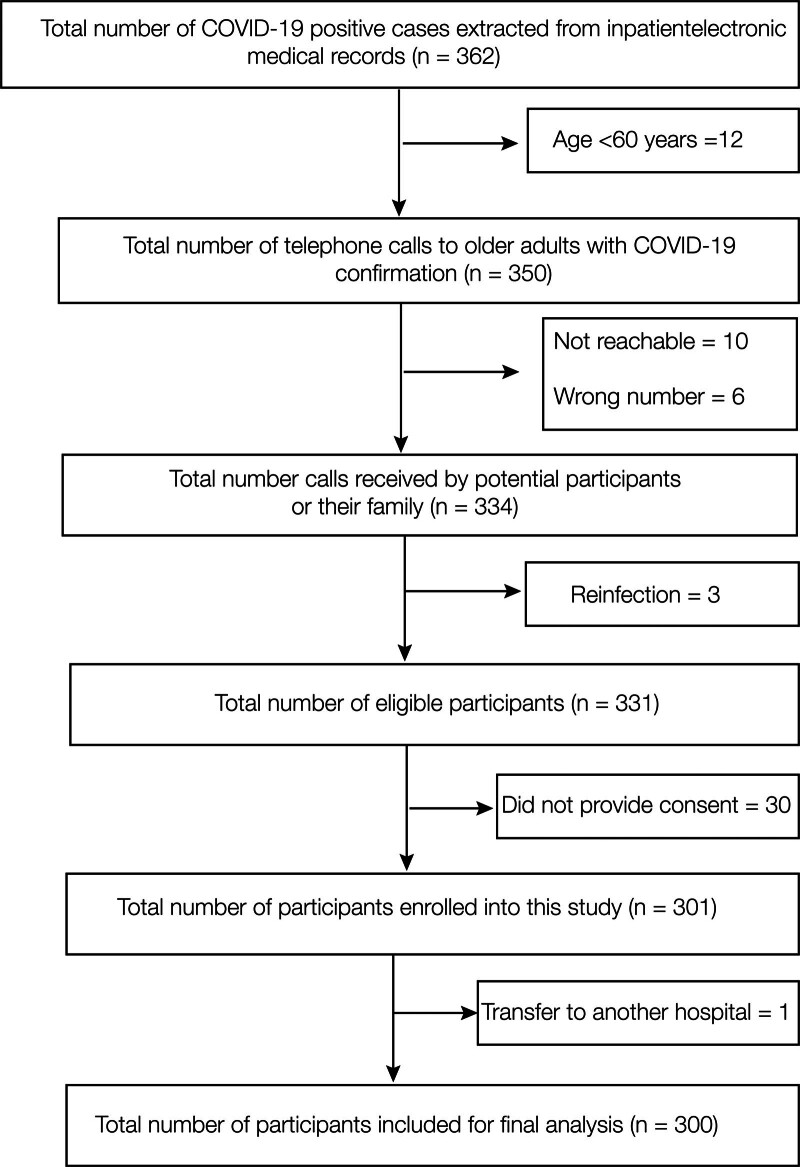
Flow chart illustrating the selection of participants enrolled in the study.

The study flowchart was presented in Figure 1. We searched inpatient medical records and eventually identified a list of 362 potential participants. We placed 350 phone calls to conduct the interviews, and received responses to 334 of those calls. By excluding individuals who did not meet the inclusion and exclusion criteria, as well as those who refused to provide consent or were transferred, we finally completed 300 interviews.

The clinical characteristics, laboratory parameters, and outcomes of the 300 participants enrolled in the study across the quartiles of the lowest level of serum albumin were shown in Table 1. Participants in the Q4 group tended to be generally younger, predominantly female, and had a higher likelihood of lower WBC, CRP, BUN, and SCR levels. Compared to other groups, fewer people are experiencing coinfection, sepsis, acute respiratory failure, shock, and death in the Q4 group. Significant differences were observed among the lowest level of serum albumin quartiles with respect to age, sex, smoking status, WBC, L, Hb, CRP, D-dimer, BUN, SCR, cerebrovascular disease, coinfection, sepsis, initial COVID-19 stage, antiviral treatment, acute respiratory failure, shock, death, and adverse outcomes (*P* < .05).

**Table 1:** Baseline characteristics of participants according to the quartiles of albumin (n = 300).

Albumin (quartile) (g/L)	Q1 (14–25)	Q2 (25.2–28.3)	Q3 (28.4–31.8)	Q4 (31.9–44.3)	*P*-value
Number	75	75	75	75	
Age (yr)	80.83 ± 9.26	82.20 ± 7.42	80.80 ± 8.80	77.21 ± 8.40	.003
Female	33 (44.00%)	21 (28.00%)	31 (41.33%)	43 (57.33%)	.004
Smoking status					
Never	52 (69.33%)	39 (52.00%)	54 (72.00%)	62 (82.67%)	.003
Quit	10 (13.33%)	12 (16.00%)	11 (14.67%)	7 (9.33%)	
Continue	13 (17.33%)	24 (32.00%)	10 (13.33%)	6 (8.00%)	
WBC (10^9^/L)	8.80 (5.32–11.34)	6.80 (4.75–9.65)	6.47 (4.59–8.66)	4.79 (3.40–6.78)	<.001
L (10^9^/L)	0.54 (0.40–0.73)	0.57 (0.48–0.79)	0.70 (0.55–1.03)	0.79 (0.59–1.28)	<.001
Hb (g/L)	115.73 ± 22.92	119.71 ± 22.95	125.04 ± 21.54	123.93 ± 14.47	.005
PLT (10^9^/L)	179 (121–218)	172 (126.5–234.5)	194 (136.5–253)	178 (130–213)	.291
CRP (g/L)	74.02 (42.60–128.20)	66.82 (32.85–122.05)	48.40 (15.99–75.12)	22.48 (5.41–51.17)	<.001
D-dimer (mg/L)	2.09 (1.04–4.59)	1.89 (0.77–4.53)	0.98 (0.59–1.89)	0.59 (0.39–0.95)	< 0.001
ALT (U/L)	23.00 (15.00–34.00)	22.00 (14.50–35.00)	24.00 (16.00–36.00)	22.00 (16.00–32.00)	.833
BUN (mmol/L)	10.30 (7.17–17.00)	9.08 (6.80–11.89)	6.55 (5.48–9.61)	5.90 (4.71–7.42)	<.001
SCR (μmol/L)	88.00 (58.05–128.00)	89.30 (69.00–126.55)	77.20 (60.55–92.85)	67.40 (60.30–88.25)	.005
CK-MB (ng/mL)	3.13 (2.26–4.87)	2.95 (1.93–4.52)	2.89 (1.80–4.16)	2.71 (2.06–3.49)	.116
Globulin (g/L)	31.60 (28.20–35.85)	30.60 (26.70–34.65)	30.60 (27.00–34.25)	30.20 (26.30–33.45)	.460
Hypertension	46 (61.33%)	55 (73.33%)	52 (69.33%)	56 (74.67%)	.280
Diabetes mellitus	28 (37.33%)	22 (29.33%)	18 (24.00%)	24 (32.00%)	.353
Chronic lung disease	19 (25.33%)	26 (34.67%)	24 (32.00%)	14 (18.67%)	.123
Cardiovascular disease	24 (32.00%)	21 (28.00%)	19 (25.33%)	19 (25.33%)	.773
Cerebrovascular disease	24 (32.00%)	19 (25.33%)	9 (12.00%)	12 (16.00%)	.012
Coinfection	60 (80.00%)	47 (62.667%)	36 (48.00%)	22 (29.33%)	<.001
Sepsis	27 (36.00%)	13 (17.33%)	2 (2.67%)	0 (0.00%)	<.001
Initial COVID-19 stage					
Moderate	31 (41.33%)	39 (52.00%)	53 (70.67%)	59 (78.67%)	<.001
Severe	13 (17.33%)	16 (21.33%)	16 (21.33%)	11 (14.67%)	
Critical	31 (41.33%)	20 (26.67%)	6 (8.00%)	5 (6.67%)	
Antiviral treatment	54 (72.00%)	53 (70.67%)	47 (62.67%)	38 (50.67%)	.025
Respiratory failure	43 (57.33%)	23 (30.67%)	8 (10.67%)	7 (9.33%)	<.001
Shock	30 (40.00%)	12 (16.00%)	3 (4.00%)	2 (2.67%)	<.001
Death	34 (45.33%)	15 (20.00%)	2 (2.67%)	1 (1.33%)	<.001
Adverse outcomes	48 (64.00%)	26 (34.67%)	7 (9.33%)	10 (13.33%)	<.001

Values are mean ± SD/median (Q1–Q3) or n (%).

Abbreviations: ALT = D-dimer, alanine aminotransferase, BUN = blood urea nitrogen, CK-MB = creatine kinase-myocardial isoenzyme, COVID-19 = coronavirus disease 2019, CRP = c-reactive protein, Hb = hemoglobin, L = lymphocyte, PLT = platelet, SCR = serum creatinine, WBC = white blood cell.

The results of univariate analysis showed that WBC, CRP, D-dimer, BUN, SCR, cardiovascular disease, and cerebrovascular disease, coinfection, sepsis, initial COVID-19 stage and antiviral treatment were positively correlated with the risk of adverse outcomes (Table 2). However, the lowest level of serum albumin was negatively correlated with adverse outcomes.

**Table 2:** The results of univariate analysis for adverse outcomes.

	Statistics	OR (95% CI)	*P*-value
Age (yr)	80.26 ± 8.66	1.017 (0.988, 1.047)	.25
Female	128 (42.67%)	0.730 (0.441, 1.209)	.221
Smoking status			
Never	207 (69.00%)	1.0	
Quit	40 (13.33%)	2.318 (1.156, 4.649)	.018
Continue	53 (17.67%)	1.583 (0.834, 3.007)	.16
WBC (10^9^/L)	6.52 (4.21–9.21)	1.082 (1.019, 1.150)	.01
L (10^9^/L)	0.64 (0.49–0.95)	0.190 (0.085, 0.427)	<.001
Hb (g/L)	121.10 ± 20.99	0.993 (0.981, 1.004)	.218
PLT (10^9^/L)	181.5 (126.75–228)	1.000 (0.997, 1.003)	.921
CRP (g/L)	52.28 (19.20–94.45)	1.011 (1.007, 1.015)	<.001
D-dimer (mg/L)	1.125 (0.59–2.60)	1.080 (1.032, 1.131)	.001
ALT (U/L)	23 (15–35)	0.999 (0.989, 1.009)	.806
BUN (mmol/L)	7.57 (5.60–11.20)	1.114 (1.063, 1.166)	<.001
SCR (μmol/L)	78.36 (60.95–109)	1.003 (1.000, 1.005)	.0388
CK-MB (ng/mL)	2.89 (1.99–4.17)	1.014 (0.979, 1.050)	.448
Albumin (g/L)	28.40 ± 4.51	0.757 (0.701, 0.819)	<.001
Globulin (g/L)	31.13 ± 5.54	1.007 (0.963, 1.053)	.752
Hypertension	209 (69.67%)	0.971 (0.569, 1.657)	.913
Diabetes mellitus	92 (30.67%)	1.347 (0.797, 2.275)	.266
Chronic lung disease	83 (27.67%)	0.986 (0.568, 1.711)	.960
Cardiovascular disease	83 (27.67%)	1.809 (1.062, 3.082)	.029
Cerebrovascular disease	64 (21.33%)	3.000 (1.694, 5.314)	<.001
Coinfection	165 (55.00%)	3.414 (1.979, 5.889)	<.001
Sepsis	42 (14.00%)	27.956 (10.483, 74.551)	<.001
Initial COVID-19 stage			
Moderate	182 (60.67%)	1.0	
Severe	56 (18.67%)	4.073 (1.843, 9.002)	<.001
Critical	62 (20.67%)	679.133 (87.873, 5248.730)	<.001
Antiviral treatment	192 (64.00%)	2.811 (1.581, 4.995)	<.001

Values are mean ± SD/median (Q1–Q3) or n (%).

Abbreviations: ALT = D-dimer, alanine aminotransferase, BUN = blood urea nitrogen, CI = confidence interval, CK-MB = creatine kinase-myocardial isoenzyme, COVID-19 = coronavirus disease 2019, CRP = c-reactive protein, Hb = hemoglobin, L = lymphocyte, OR = odds ratio, PLT = platelet, SCR = serum creatinine, WBC = white blood cell.

Multivariate regression analyses were performed and the results were presented in Table 3. The lowest level of serum albumin was negatively correlated with adverse outcomes after adjusting potential confounders (OR = 0.827, 95% CI = 0.706, 0.969, *P* = .019). Additionally, the lowest levels of serum albumin as a categorical variable (quartiles) were also analyzed and the trend test remained significant (*P* = .011).

**Table 3:** Relationship between the lowest level of albumin during hospitalization and adverse outcomes.

Exposure	OR (95% CIs)	*P*-value
Albumin, g/L	0.827 (0.706, 0.969)	.019
Albumin (quartile), g/L		
Q1	Ref	Ref
Q2	0.065 (0.011, 0.365)	.002
Q3	0.014 (0.002, 0.122)	.0001
Q4	0.172 (0.033, 0.894)	.036
*P* for trend		.011

Age, sex, smoking status, hypertension, diabetes mellitus, chronic lung disease, cardiovascular disease, cerebrovascular disease, coinfection, sepsis, initial COVID-19 stage, WBC, L, CRP, D-dimer, BUN, SCR, globulin, and antiviral treatment were adjusted.

Abbreviations: BUN = blood urea nitrogen, CI = confidence interval, COVID-19 = coronavirus disease 2019, CRP = C-reactive protein, L = lymphocyte, OR = odds ratio, SCR = serum creatinine, WBC = white blood cell.

**Figure 2.**
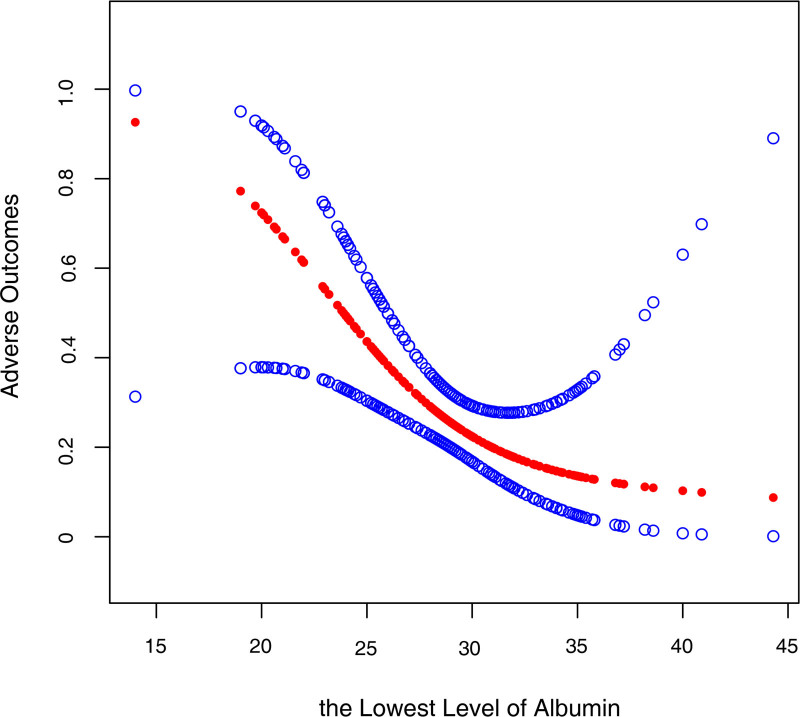
Association between the lowest value of albumin during hospitalization and adverse outcomes. A threshold, nonlinear association between albumin and adverse outcomes was found (*P* = .001) in a generalized additive model after adjusting for age, sex, smoking status, hypertension, diabetes mellitus, chronic lung disease, cardiovascular disease, cerebrovascular disease, coinfection, sepsis, initial COVID-19 stage, WBC, L, CRP, D-dimer, blood urea nitrogen, SCR, globulin, and antiviral treatment. The solid red line represents the smooth curve fit between variables. Bule dashed lines represent the 95% CI from the fit. COVID-19 = coronavirus disease 2019, CRP = C-reactive protein, L = lymphocyte, WBC = white blood cell.

The nonlinear relationship between the lowest level of serum albumin and adverse outcomes was detected after adjusting for potential confounders (Fig. 2). The inflection point of the lowest level of serum albumin was 29.1 g/L calculated by using a 2-piecewise linear regression model (Table 4). The effect sizes and the confidence intervals on the left and right sides of inflection point were 0.667 (0.520, 0.856) and 1.171 (0.875, 1.568), respectively. This demonstrated when the lowest level of serum albumin during hospital stay was <29.1 g/L, for every 1 unit increase in serum albumin, the risk of adverse outcomes was reduced by 33.3%.

**Table 4:** The results of the 2-piecewise linear regression model.

Albumin (g/L)	Odds ratio (95% CIs)	*P*-value
≤29.1	0.667 (0.520, 0.856)	.001
>29.1	1.171 (0.875, 1.568)	.289

Adjusted for age, sex, smoking status, hypertension, diabetes mellitus, chronic lung disease, cardiovascular disease, cerebrovascular disease, coinfection, sepsis, initial COVID-19 stage, WBC, L, CRP, D-dimer, BUN, SCR, globulin, and antiviral treatment.

Abbreviations: BUN = blood urea nitrogen, CI = confidence interval, COVID-19 = coronavirus disease 2019, CRP = C-reactive protein, L = lymphocyte, SCR = serum creatinine, WBC = white blood cell.

## 4. Discussion

This study elucidated that the lowest level of serum albumin during hospitalization, both as a continuous variable and as a categorical variable by quartiles, was negatively correlated with the risk of adverse outcomes, as shown in multivariate regression analysis. However, further analysis showed that the relationship between them was nonlinear and the inflection point of the lowest level of serum albumin during hospitalization was 29.1 g/L. No significant difference was found on the right side of the inflection point, but negative association between the lowest level of serum albumin during hospitalization and adverse outcomes was observed on the left of the inflection point. It means that when the lowest level of serum albumin is <29.1 g/L, a rise of 1 unit in the lowest level of serum albumin during hospitalization leads to a 33.3% reduction in the risk of experiencing adverse outcomes.

Albumin, a major plasma protein, is synthesized in the liver and has a serum half-life of approximately 21 days.^[15]^ It influences both innate and adaptive immune responses, serves as a transport molecule, interacts with bioactive lipid mediators, prevents oxidative damage, stabilizes the endothelium, and controls plasma osmotic pressure.^[16,17]^ Nonetheless, serum albumin levels significantly decrease in the acute inflammatory phase, approximately by 50%.^[18]^ Multiple mechanisms contributed to the reduction of serum albumin in older patients. Firstly, severe inflammatory response in COVID-19 may lead to increased capillary permeability and leakage of albumin into interstitial space.^[19,20]^ Secondly, the systemic inflammatory response can also accelerate albumin catabolism, decrease the synthesis of albumin, and downregulate hepatic secretion of albumin.^[21,22]^ Thirdly, serum albumin concentrations in the general population peak at about 20 years of age, and then declines with age.^[4]^

Previous researches have reported that decline of albumin on admission is a predictor of adverse outcomes,^[11,13,23–25]^ while a higher albumin level at admission predicts a better prognosis for COVID-19 patients.^[12]^ Additionally, Xie et al identified the turning point of serum albumin levels; however, their study population ranged in age from 16 to 93 years, and the serum albumin measured was the initial albumin level upon admission.^[26]^ However, it is unlikely for clinicians to alter the initial albumin levels at admission. In comparison, the lowest albumin levels detected in COVID-19 patients can be relatively easily elevated above the threshold when necessary.

Critically ill patients may benefit from albumin infusion. Zhang et al reported that compared with control patients, patients who received albumin infusion therapy survived significantly longer and had a shorter hospital stay.^[27]^ Another study highlighted the impact of albumin infusion on hypercoagulability in patients hospitalized with SARS-CoV-2 infection.^[28]^ Twenty-nine pneumonia patients with COVID-19 (D-dimer levels > 1 μg/mL and serum albumin < 3.5 g/dL) were divided into 2 groups. Ten patients with a mean age of 82 years received albumin infusions for 7 days, while 19 with a mean age of 73 years served as control. Both groups received low-dose heparin. Albumin was administered at 80 g/day for the first 3 days and 40 g/day for the next 4 day. In the albumin group, serum albumin levels increased from 2.7 to 3.6 g/dL, and D-dimer levels decreased from 3.23 to 1.3 μg/mL. In the control group, serum albumin levels slightly decreased from 3.0 to 2.9 g/dL, and D-dimer levels increased from 3.37 to 4.4 μg/mL. One hemorrhagic event was observed in the albumin group, while the control group experienced 4 deaths, 2 cases of ischemia, 1 pulmonary embolism, and 1 stroke. This study suggests that albumin may exert an anticoagulatory activity by decreasing the plasma level of D-dimer. Albumin infusion combined with conventional anti-aggregators may be the optimal anticoagulant therapy for critically ill patients with or without COVID-19.^[7]^

Moreover, albumin infusions within the first 24 hours or during the first week has been shown to elevate blood pressure in patients experiencing shock.^[29]^ Additionally, albumin infusion in human septic shock has a nephroprotective potential by inhibit heparin-binding-protein-induced endothelial cell permeability.^[30]^ Serum albumin is a crucial indicator of nutritional status in cancer patients and closely linked to their cancer prognosis. Furthermore, the role of recombinant albumin and albumin-based nanocarriers in drug delivery and cancer therapy is the focus of extensive research.^[31,32]^

However, numerous conditions are considered unsuitable for albumin infusion due to the lack of significant benefit.^[33]^ The threshold effect identified in this study may elucidate the observed ineffectiveness of albumin infusion in these scenarios. In addition to the dosage of albumin infusion, appropriate opportunity of albumin administration also requires further investigation. As underscored by Singh et al, albumin supportive therapy could potentially prevent the progression to severe COVID-19.^[34]^ They further pointed out that late supplementation of albumin after organ failure had no benefit.^[34]^

In this study, some limitations are found. First, due to the retrospective nature of the study, the association between exposure and outcome is weak. Second, as the study population contains only older patients with COVID-19, the findings of the study apply exclusively to the elderly and may not be generalized to all demographic groups. Third, almost all elderly individuals are likely to have a high prevalence of underlying conditions, which could serve as confounding factors and potentially influence the conclusions of real-world studies. Therefore, future research should employ more advanced diagnostic techniques to identify these underlying conditions.

## 5. Conclusion

This research revealed a nonlinear relationship between the lowest value of albumin during hospitalization and adverse outcomes in the elderly with COVID-19. The lowest value of albumin is negatively correlated with adverse outcomes when the lowest value of albumin is <29.1 g/L. These results may help clinicians identify underlying patients with poor outcomes, and take precautions against adverse outcomes.

## Acknowledgments

We would like to express our gratitude for the guidance in statistical analysis provided by Professor Chi Chen.

## Author contributions

**Conceptualization:** Qiaoli Liu, SuHong An.

**Data curation:** Haifeng Miao, Chunwei Shi.

**Formal analysis:** SuHong An.

**Investigation:** Piao Hu.

**Methodology:** Haifeng Miao.

**Resources:** Qiaoli Liu.

**Software:** Chunwei Shi.

**Supervision:** Piao Hu.

**Writing – original draft:** Qiaoli Liu.
